# Climate driven spatiotemporal variations in seabird bycatch hotspots and implications for seabird bycatch mitigation

**DOI:** 10.1038/s41598-021-00078-z

**Published:** 2021-10-19

**Authors:** Rujia Bi, Yan Jiao, Joan A. Browder

**Affiliations:** 1 Department of Fish and Wildlife Conservation, Virginia Polytechnic Institute and State University, Virginia Tech, Blacksburg, VA 24061 USA; 2 NOAA National Marine Fisheries Service, Southeast Fisheries Science Center, Miami, FL 33149 USA

**Keywords:** Conservation biology, Statistics

## Abstract

Bycatch in fisheries is a major threat to many seabird species. Understanding and predicting spatiotemporal changes in seabird bycatch from fisheries might be the key to mitigation. Inter-annual spatiotemporal patterns are evident in seabird bycatch of the U.S. Atlantic pelagic longline fishery monitored by the National Marine Fisheries Service Pelagic Observer Program (POP) since 1992. A newly developed fast computing Bayesian approximation method provided the opportunity to use POP data to understand spatiotemporal patterns, including temporal changes in location of seabird bycatch hotspots. A Bayesian model was developed to capture the inherent spatiotemporal structure in seabird bycatch and reduce the bias caused by physical barriers such as coastlines. The model was applied to the logbook data to estimate seabird bycatch for each longline set, and the mid-Atlantic bight and northeast coast were the fishing areas with the highest fleet bycatch estimate. Inter-annual changes in predicted bycatch hotspots were correlated with Gulf Stream meanders, suggesting that predictable patterns in Gulf Stream meanders could enable advanced planning of fishing fleet schedules and areas of operation. The greater the Gulf Stream North Wall index, the more northerly the seabird bycatch hotspot two years later. A simulation study suggested that switching fishing fleets from the hindcasted actual bycatch hotspot to neighboring areas and/or different periods could be an efficient strategy to decrease seabird bycatch while largely maintaining fishers’ benefit.

## Introduction

A large proportion of seabird species are in decline^1^. The most recent global assessment revealed that 31% of all seabird species are globally Threatened and another 11% are Near Threatened by International Union for Conservation of Nature (IUCN) Red List criteria^1,2^; incidental mortality associated with fisheries has been recognized as a key threat^2–5^. The situation is more severe for species with a broad foraging range, tendency to follow vessels, late maturity and low reproduction rate, such as the Procellariiformes including albatrosses, petrels, fulmars and shearwaters^6–9^. Longline fisheries, in which seabirds may swallow hooks or be entangled in lines, can be a major cause of bycatch and are monitored to assess bycatch magnitude^10,11^. The imperative to assess the impact of longline fisheries on seabird populations and develop management actions to reduce the corresponding seabird bycatch is well recognized, and studies on total longline fishery bycatch, fishing practices, bird life history and spatial factors that influence seabird bycatch have been ongoing^9,12,13^. Comprehensive research established efficient mitigation measures such as weighted lines, bird-scaring lines, blue-dyed baits and night setting for longline fisheries, and these measures have been demonstrated to reduce seabird bycatch effectively^14–16^. Recent studies have generated questions about bycatch variation in space and time and how and why locations of high seabird bycatch (i.e., “hotspots”) change inter-annually. Answers to these questions may enable better mitigation of bycatch.

**Figure 1:**
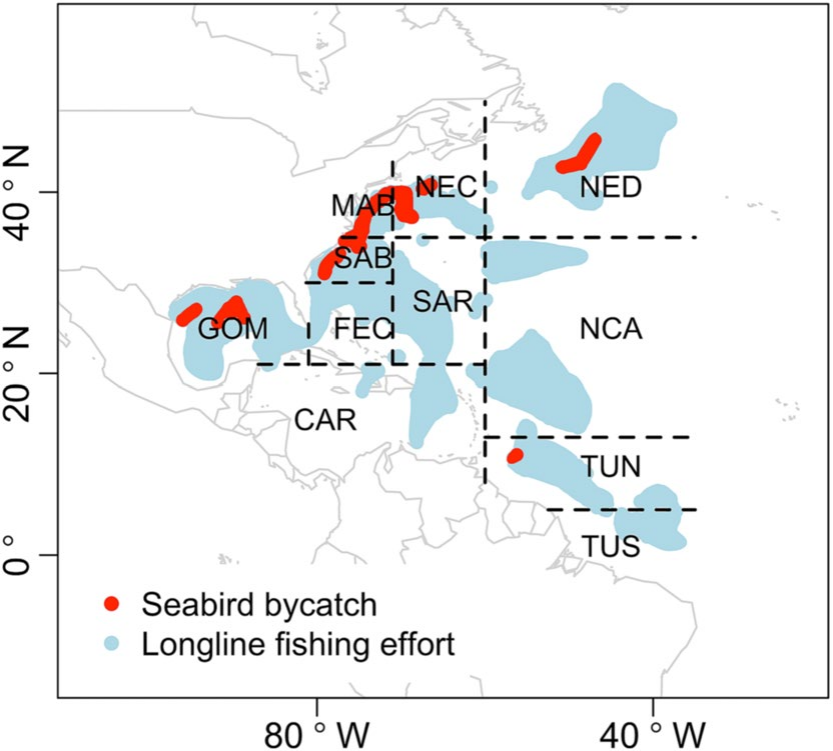
Spatial distribution of observed longline sets (blue area) and those with seabirds caught (red strips) in 11 fishing zones from 1992 to 2017. Abbreviations represent the following: NED – Northeast district, NCA – North Central Atlantic, TUN – Tuna North, TUS – Tuna South, NEC – Northeast coast, SAR – Sargasso region, CAR – Caribbean region, MAB – Mid-Atlantic bight, SAB – South Atlantic bight, FEC – Florida east coast, GOM – Gulf of Mexico. Plot is made using R package ggplot2 (version 3.3.2, https://ggplot2.tidyverse.org) in statistical program R (version 3.6.3, http://www.R-project.org/). Map data is from R package maps (version 3.3.0, https://CRAN.R-project.org/package=maps).

To meet the need of assessing bycatch from the longline fishery, the National Oceanic and Atmospheric Administration (NOAA) National Marine Fisheries Service Southeast Fisheries Science Center (SEFSC) has been monitoring the western North Atlantic U.S. pelagic longline fishery through the Pelagic Observer Program (POP) since 1992^17,18^. The fishery operates in 11 specified fishing zones (Fig. 1) and targets tuna (*Thunnus* spp.), swordfish (*Xiphias gladius*), dolphinfish (*Coryphaena hippurus*), and pelagic sharks (various Selachimorpha)^19^. The SEFSC POP collects effort, catch and bycatch (including seabirds) information from randomly selected fishing trips with a coverage rate of about 8% of the trips in each fishing zone and each calendar quarter^18^. The POP data can be used to identify factors influencing bycatch rate, predict the seabird bycatch in the pelagic longline fishery and provide information to develop management plans.

There are two inherent features of the POP data that make the assessment of seabird bycatch in the U.S. Atlantic pelagic longline fishery challenging. First, seabird bycatch is a rare event, with most counts by set being zero. Second, bycatch data are spatially autocorrelated, particularly in how the bycatch event is geographically referenced. Hurdle models have been proposed to deal with the excessive number of zeroes in the data. This type of model consists of two components: a Bernoulli component that models the probability of producing a bycatch event and a zero-truncated component that models the positive records^12,20–24^. Spatial filters, spatial expansion and geographically weighted regressions have been used to consider spatial autocorrelations of seabird bycatch^12,13^. Bayesian hierarchical modelling provides a flexible framework to incorporate spatiotemporal trends by treating spatial and temporal variables as random effects^25–27^. Traditional Markov Chain Monte Carlo (MCMC) algorithms can require a long computational time when dealing with a continuous spatial field^28^. As an alternative, a new statistical approach addresses the computational challenges in Bayesian modelling by approximating the marginal posterior using integrated nested Laplace approximations (INLA) methodology^29–31^. In particular, INLA implements the stochastic partial differential equations (SPDE) approach, which provides an effective solution to simulate a spatial effect^32^. The model fitted through the INLA-SPDE approach can capture spatial autocorrelation in data in a short computational time^29–32^. With multiple years of data on seabird bycatch from a pelagic longline fishery and the fast INLA computation, the prediction of inter-annual variation of central major bycatch areas and their possible linkage to climate changes became practical.

Our objectives were to develop Bayesian hierarchical hurdle models using the 1992–2017 POP data to 1) identify the inter-annual hotspots of seabird bycatch, 2) diagnose whether inter-annual hotspot variation is supported by data and whether the variation is linked to monitored and predictable oceanic or climate cycles, 3) predict seabird bycatch in the U.S Atlantic pelagic longline fishery by extrapolating from the logbook data, and 4) explore the potential effectiveness of seabird bycatch mitigation measures based on forecasts of hotspot locations and seasons with simulation experiments. Given the complex coastlines in our study region, we extended the commonly used stationary SPDE approach to non-stationary spatial fields to reduce the bias caused by physical barriers^33^. The indices used to describe oceanic/climate variations included the winter (i.e., December-March mean) North Atlantic Oscillation (NAO) index^34^, annual Atlantic Multidecadal Oscillation (AMO) index^35^, and annual Gulf Stream North Wall (GSNW) index, a metric of the latitudinal position of the Gulf Stream as it separates from the U.S. coast near Cape Hatteras and travels eastwards across the North Atlantic as meanders (personal communication with Dr. Arnold H. Taylor at Plymouth Marine Laboratory). These indices were evaluated to interpret inter-annual hotspot variation.

## Results

### Model comparison and selected explanatory variables

For data from all observer coverage areas, the bycatch probability sub-model fitted with a spatial effect that was constant over time (model M7) performed better than the others, according to the smaller deviance information criterion (DIC)^36^ and Watanabe-Akaike information criterion (WAIC)^37^ values in Table 1. Incorporation of a spatial field into the positive bycatch sub-model did not improve model fit to data.

**Table 1:** Models fitted to data from 11 fishing zones. Abbreviations are as follow: DIC_z_ – the deviance information criterion (DIC) value on the bycatch probability component, DIC_y_ – the DIC value on the positive bycatch component, WAIC_z_ – the Watanabe-Akaike information criterion (WAIC) value on the bycatch probability component, WAIC_y_ – the WAIC value on the positive bycatch component, $\xi_{z}$ and $\xi_{y}$ – constant spatial effect; $\xi_{z}^{t}$ and $\xi_{y}^{t}$ – spatial effect with a different realization every year. Top performing models in each step were listed for brevity.

Model	Probability	Positive bycatch	DIC_z_	DIC_y_	WAIC_z_	WAIC_y_
M0	Intercept	Intercept	1174.01	260.78	1174.01	262.48
M1	Intercept + water temperature	Intercept + number of hooks	1098.06	252.39	1098.28	258.45
M2	Intercept + water temperature + season	Intercept + number of hooks + haul time	1070.52	234.82	1070.33	241.70
M3	Intercept + water temperature + season + target species	Intercept + number of hooks + haul time	1047.66	234.83	1047.49	241.70
M4	Intercept + water temperature + season + target species + year	Intercept + number of hooks + haul time	1034.74	234.98	1032.31	241.79
M5	Intercept + water temperature + season + target species + year + set time	Intercept + number of hooks + haul time	1028.69	235.02	1026.09	241.81
M6	Intercept + water temperature + season + target species + year + set time + vessel ID	Intercept + number of hooks + haul time	1015.87	234.89	988.33	241.74
M7	Intercept + water temperature + season + target species + year + set time + vessel ID + $\xi_{z}$	Intercept + number of hooks + haul time	995.06	234.88	975.29	241.73

For data from the three high-bycatch zones (northeast coast, NEC, 60–71°W, 35–42°N; mid-Atlantic bight, MAB, 71–82°W, 35–41°N; and south Atlantic bight, SAB, 71–82°W, 30–35°N; Fig. 1), the bycatch probability sub-model fitted with time-varying spatial effect (model T7) performed better (Table 2); the positive bycatch sub-model fitted with the spatial effect did not improve model performance significantly. Therefore, model T7 was selected when fitting to data from the three high-bycatch zones.

**Table 2:** Models fitted to data from the three high-bycatch zones. Abbreviations are as follow: DIC_z_ – the deviance information criterion (DIC) value on the bycatch probability component, DIC_y_ – the DIC value on the positive bycatch component, WAIC_z_ – the Watanabe-Akaike information criterion (WAIC) value on the bycatch probability component, WAIC_y_ – the WAIC value on the positive bycatch component, $\xi_{z}$ and $\xi_{y}$ – constant spatial effect; $\xi_{z}^{t}$ and $\xi_{y}^{t}$ – spatial effect with a different realization every year. Top performing models in each step were listed for brevity.

Model	Probability	Positive bycatch	DIC_z_	DIC_y_	WAIC_z_	WAIC_y_
T0	Intercept	Intercept	837.45	236.61	837.45	238.20
T1	Intercept + year	Intercept + number of hooks	802.54	222.57	799.62	229.43
T2	Intercept + year + season	Intercept + number of hooks + haul time	774.33	216.38	770.57	223.15
T3	Intercept + year + season + set time	Intercept + number of hooks + haul time	754.51	216.42	750.57	223.17
T4	Intercept + year + season + set time + water temperature	Intercept + number of hooks + haul time	739.53	216.46	735.20	223.20
T5	Intercept + year + season + set time + water temperature + vessel ID	Intercept + number of hooks + haul time	732.76	216.46	727.81	223.20
T6	Intercept + year + season + set time + water temperature + vessel ID + target species	Intercept + number of hooks + haul time	727.12	216.39	721.86	223.15
T7	Intercept + year + season + set time + water temperature + vessel ID + target species + $\xi_{z}^{t}$	Intercept + number of hooks + haul time	716.54	216.59	710.33	223.29

**Figure 2:**
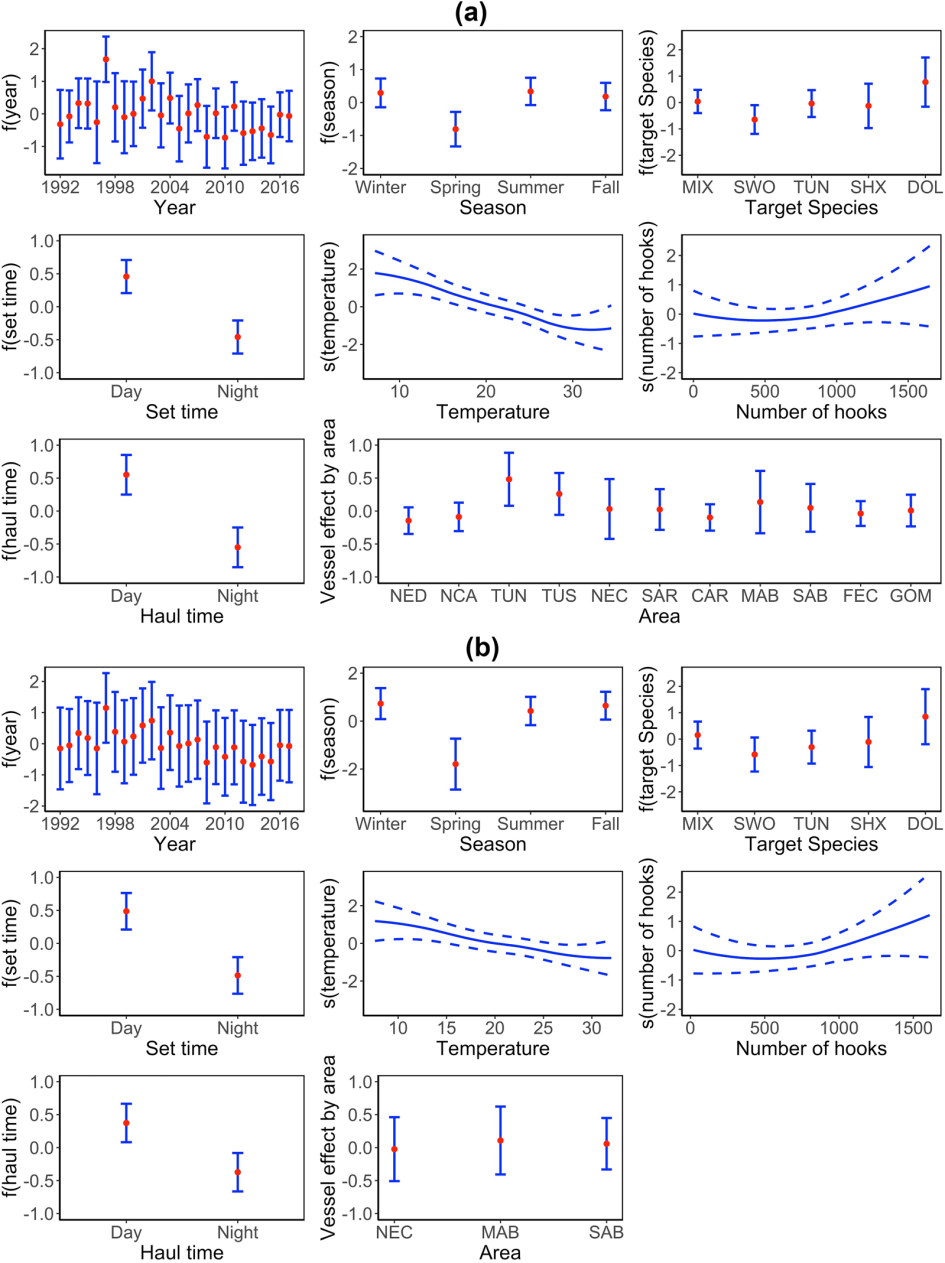
(**a**) Impacts of year, season, target species, set time and water temperature on logit(*p*), impacts of number of hooks and haul time on ln(positive number of seabird bycatch per longline set), vessel effects on logit(*p*) grouped by area from model M7 fitted with data from 11 fishing zones. (**b**) Impacts of year, season, target species, set time and water temperature on logit(*p*) and impacts of number of hooks and haul time on ln(positive number of seabird bycatch per longline set), vessel effects on logit(*p*) grouped by area from model T7 fitted to data from the three high-bycatch zones. Points and solid lines represent posterior mean values; error bars and dashed lines represent 95% credible intervals. For the area-average vessel effects, points represent area-specific mean of the posterior means and error bars represent standard deviations of the posterior means. Abbreviations of target species are as follow: MIX – Mixed species, SWO – Swordfish, TUN – Tuna, SHX – Shark, DOL – Dolphinfish. Abbreviations of fishing zones are as follow: NED – Northeast district, NCA – North Central Atlantic, TUN – Tuna North, TUS – Tuna South, NEC – Northeast coast, SAR – Sargasso region, CAR – Caribbean region, MAB – Mid-Atlantic bight, SAB – South Atlantic bight, FEC – Florida east coast, GOM – Gulf of Mexico. Plot is made using R package ggplot2 (version 3.3.2, https://ggplot2.tidyverse.org) in statistical program R (version 3.6.3, http://www.R-project.org/).

**Figure 3:**
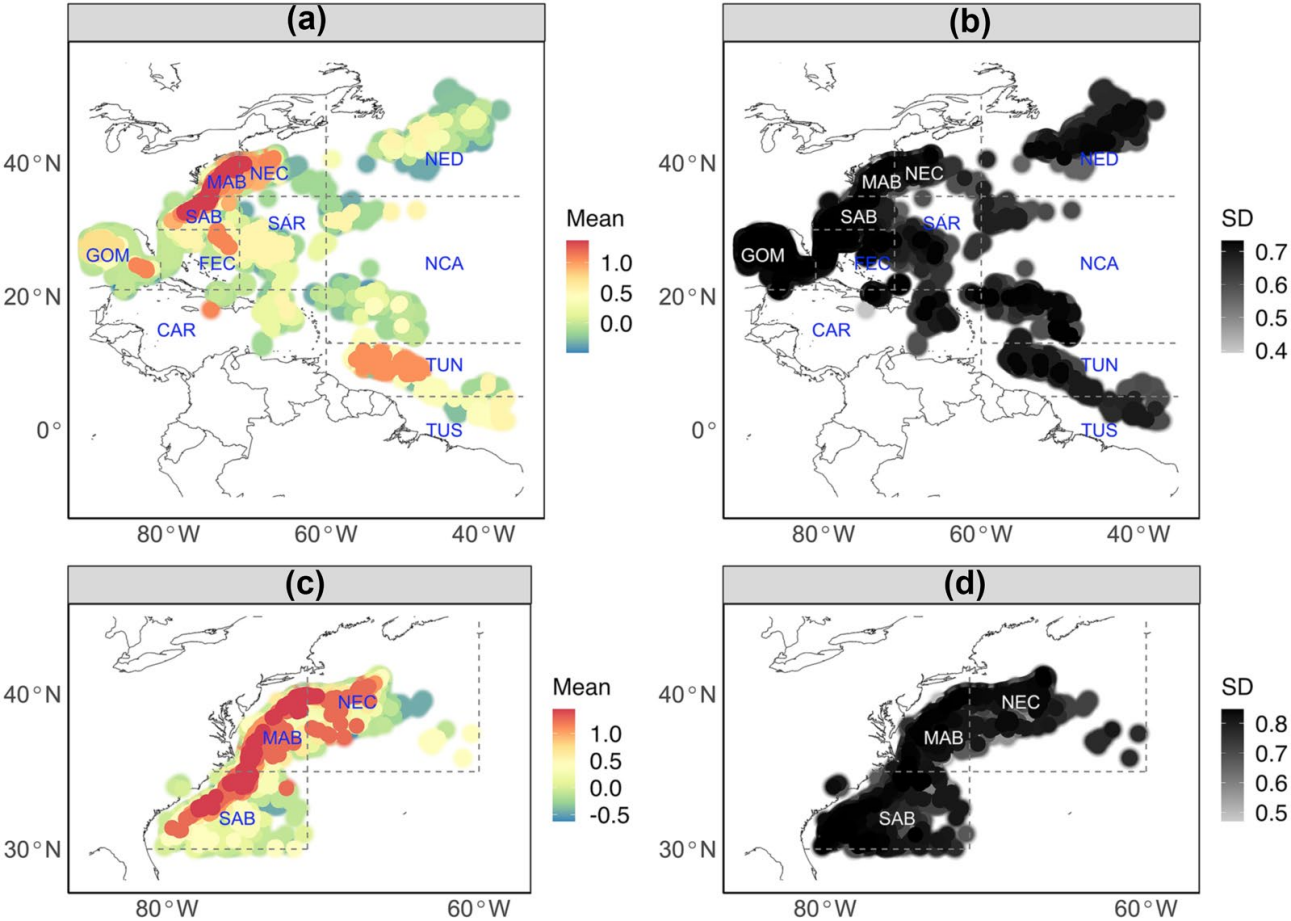
(**a,b**) Posterior mean and standard deviation of impacts of vessel ID on logit(*p*) from model M7 fitted with data from 11 fishing zones. (**c,d**) Posterior mean and standard deviation of impacts of vessel ID on logit(*p*) from model T7 fitted with data from the three high-bycatch zones. Vessel position is determined by longitude and latitude of start-setting location. Large and blurred points indicate approximate vessel positions because of the confidential concerns. Vessels with greater effects on logit(*p*) are plotted on the top. Plot is made using R package ggplot2 (version 3.3.2, https://ggplot2.tidyverse.org) in statistical program R (version 3.6.3, http://www.R-project.org/). Map data is from R package maps (version 3.3.0, https://CRAN.R-project.org/package=maps).

The effects of year, season, target species, water temperature, set time and vessel ID on logit-transformed probability of catching a seabird, i.e. logit(*p*), and the effect of number of hooks and haul time on positive seabird bycatch rate are shown in Fig. 2. The year effect on logit(*p*) showed clear inter-annual variations and peaked around 1997. Most of the seabird bycatch was estimated to occur during summer through winter. Longline sets targeting dolphinfish were estimated to have the highest seabird bycatch probability; while longline sets targeting mixed species, sharks and tuna had relatively intermediate probabilities, and longline sets targeting swordfish had the lowest seabird bycatch probability. Daytime setting was associated with higher bycatch probability compared to night setting. There was a negative relationship between water temperature and seabird bycatch probability. There was a positive relationship between number of hooks and seabird bycatch rate. Daytime haul-back was associated with greater seabird bycatch rate. The mean vessel effects grouped by area suggested a greater effect in the Tuna North (TUN) area. A map of the vessel ID effects is displayed in Fig. 3. Some vessels fishing at the MAB, SAB, NEC and TUN areas were associated with higher seabird bycatch probability.

### Hotspots of observed seabird bycatch and their correlations with climate variations

**Figure 4:**
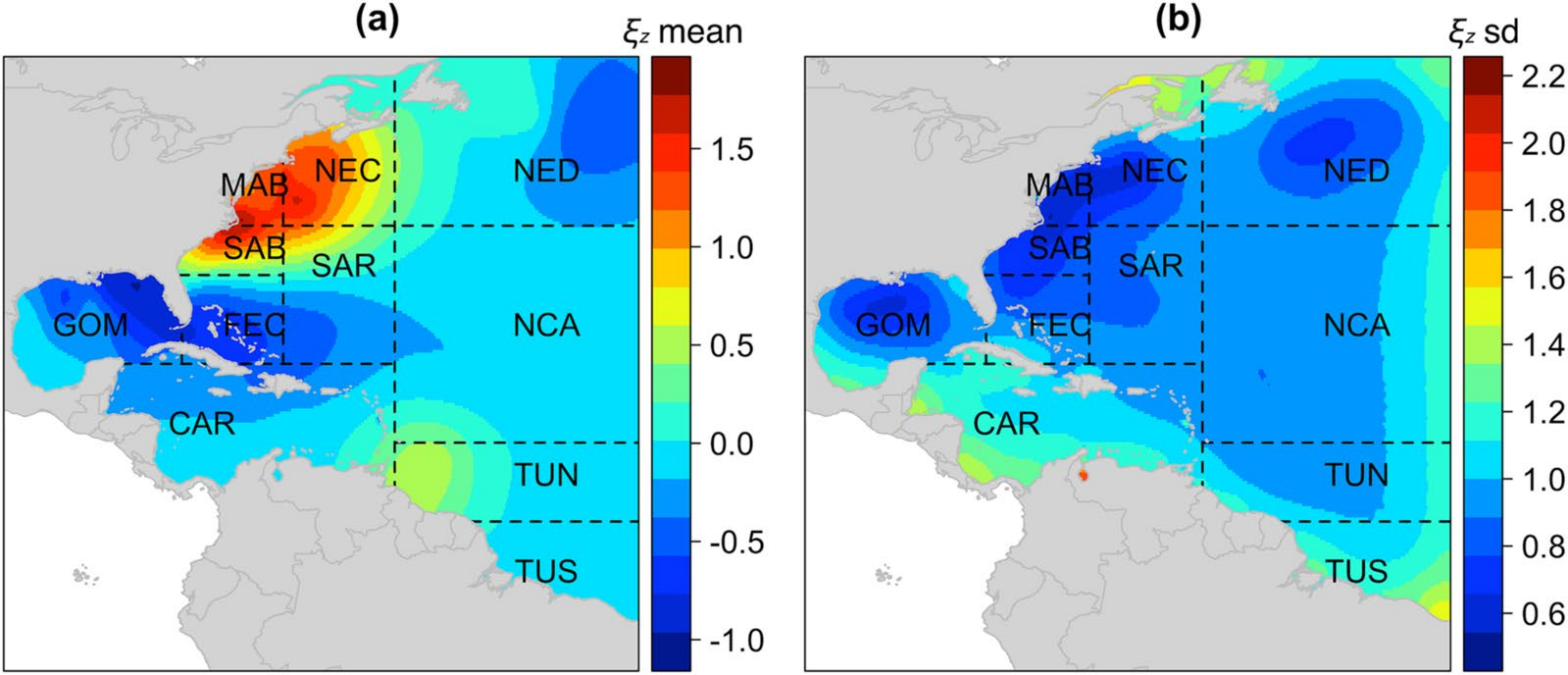
(**a**) Posterior mean and (**b**) standard deviation of the spatial random field on logit(*p*) from model M7 fitted to data from 11 fishing zones. Abbreviations represent the following: NED – Northeast district, NCA – North Central Atlantic, TUN – Tuna North, TUS – Tuna South, NEC – Northeast coast, SAR – Sargasso region, CAR – Caribbean region, MAB – Mid-Atlantic bight, SAB – South Atlantic bight, FEC – Florida east coast, GOM – Gulf of Mexico. Plot is made using R package lattice (version 0.20–38, http://lmdvr.r-forge.r-project.org) in statistical program R (version 3.6.3, http://www.R-project.org/). Map data is from R package maps (version 3.3.0, https://CRAN.R-project.org/package=maps).

For all observer coverage areas, i.e. the 11 fishing zones shown in Fig. 1, the mean and standard deviation of the spatial effect on logit(*p*) are shown in Fig. 4. High bycatch probability occurred around the MAB, SAB, NEC areas (Fig. 4a). The pattern of uncertainty was driven by the amount of information. The uncertainty estimate revealed relatively low uncertainty along the coastline, where most observed longline operations occurred, and higher uncertainty farther away from the coastline (Fig. 4b).

**Figure 5:**
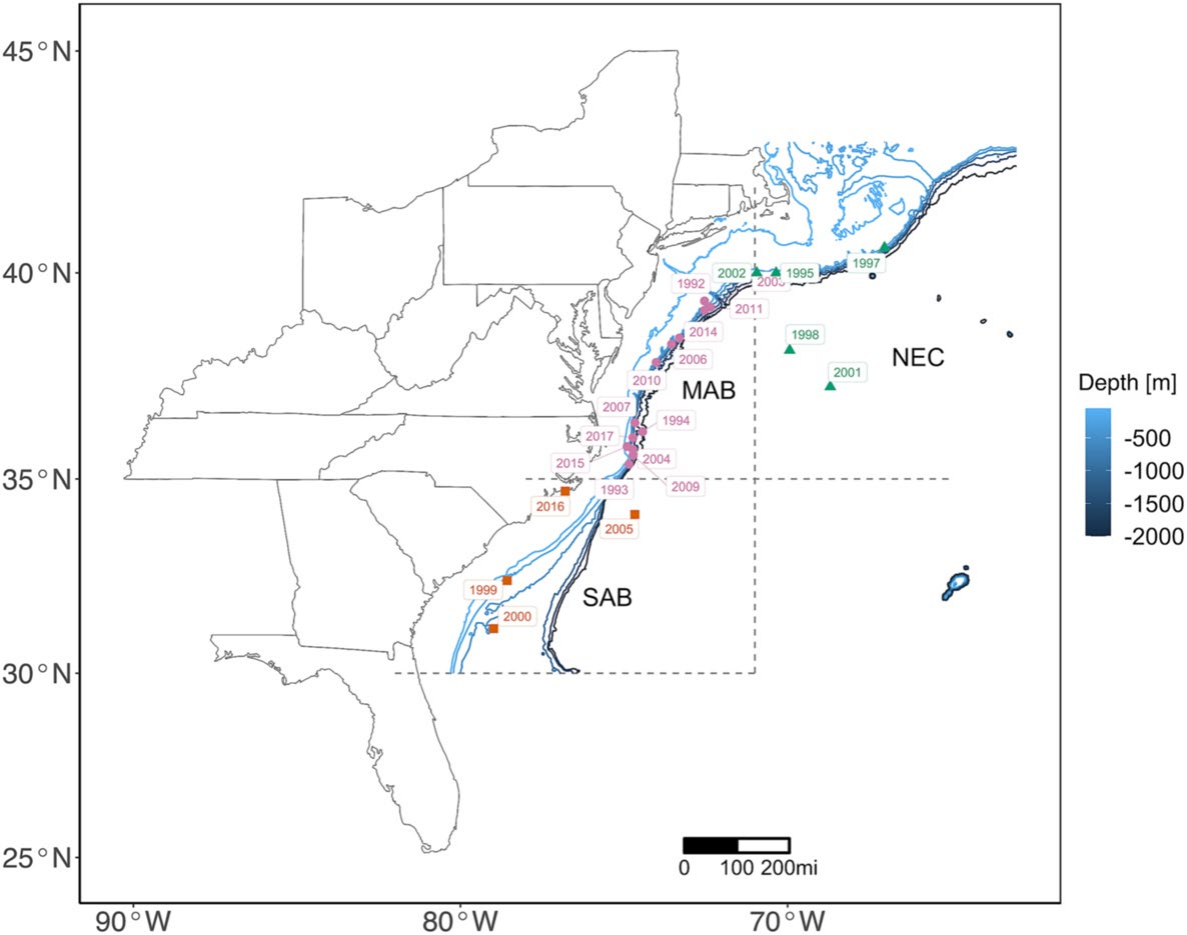
Annual seabird bycatch hotspot location from model T7 fitted to data from the three high-bycatch zones. Years with zero seabird caught (i.e. 1996, 2008, 2012 and 2013) are not shown. Hotspot locations in different fishing zones are displayed with different colors and shapes. Plot is made using R package ggplot2 (version 3.3.2, https://ggplot2.tidyverse.org) in statistical program R (version 3.6.3, http://www.R-project.org/). Map data is from R package maps (version 3.3.0, https://CRAN.R-project.org/package=maps). Bathymetry data is from R package marmap (version 1.0.5, https://github.com/ericpante/marmap).

For the three East Coast high-bycatch zones, the spatial effect differed from year to year, with bycatch hotspots located in the MAB in most years (Fig. 5). Most bycatch hotspots in the MAB were located on the shelf break, where pelagic seabirds clustered. This was not the case in regard to hotspots in the SAB and NEC areas (e.g., hotspots in 1998, 2001, 2005 and 2016). Cross-correlation analyses^38^ performed to detect the relationships between the location of the hotspot of seabird bycatch and long-term climate oscillations suggested the GSNW index of two years past displayed a significant positive effect on the latitude of the seabird bycatch hotspot in the three high-bycatch zones (*p* < 0.05); that is, the greater the GSNW, the more northerly the hotspot two years later.

### Seabird bycatch estimates from the U.S. Atlantic pelagic longline fishery

**Figure 6:**
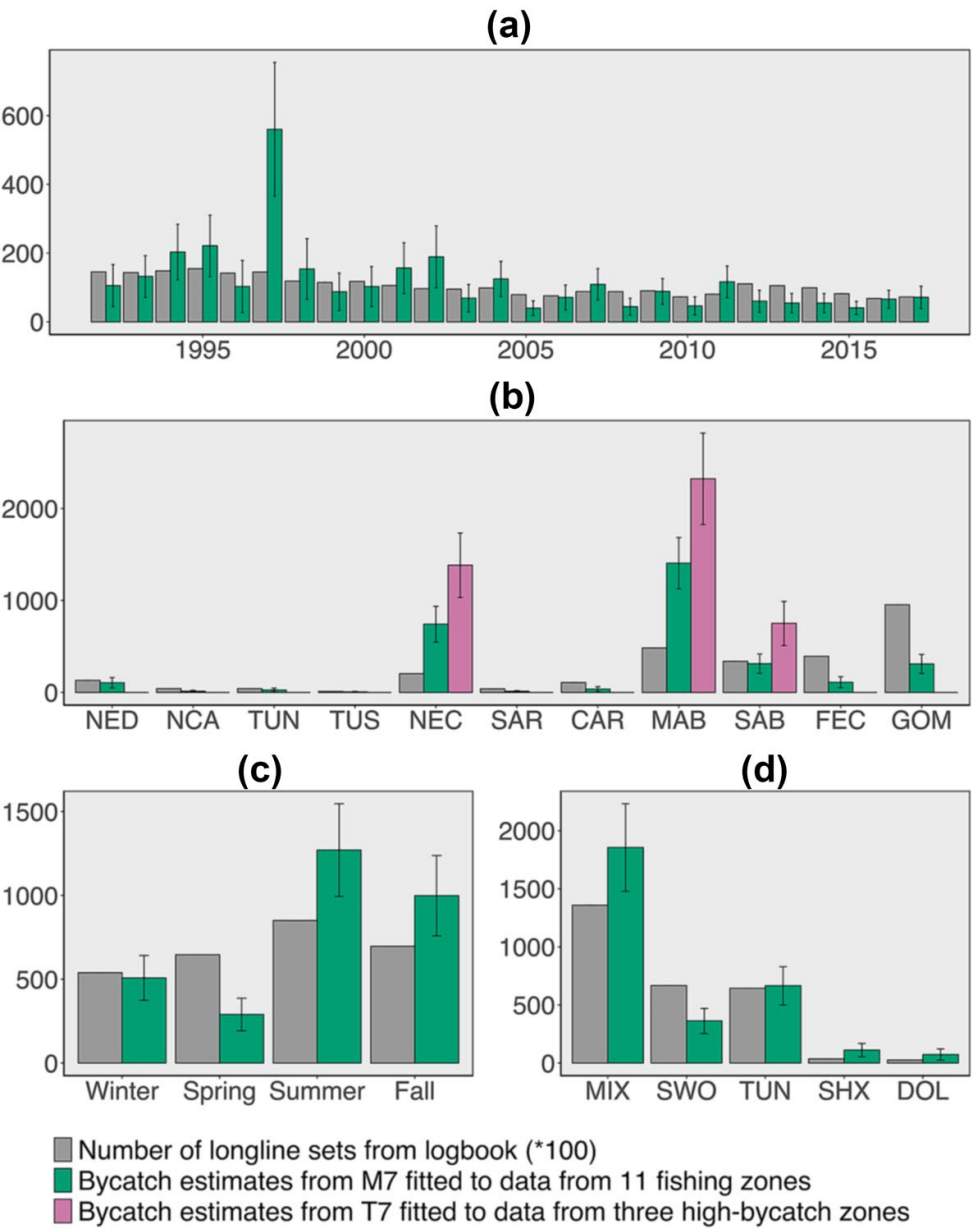
(**a**) Annual number of longline sets from logbook and seabird bycatch estimates (in number) for all 11 fishing areas from 1992 to 2017 from model M7 fitted to data from 11 fishing zones. (**b**) Number of longline sets from logbook and seabird bycatch estimates (in number) by area from 1992 to 2017. Abbreviations represent the following: NED – Northeast district, NCA – North Central Atlantic, TUN – Tuna North, TUS – Tuna South, NEC – Northeast coast, SAR – Sargasso region, CAR – Caribbean region, MAB – Mid-Atlantic bight, SAB – South Atlantic bight, FEC – Florida east coast, GOM – Gulf of Mexico. (**c**) Number of longline sets from logbook and seabird bycatch estimates (in number) by season for all 11 fishing areas from 1992 to 2017 from model M7 fitted to data from 11 fishing zones. (**d**) Number of longline sets from logbook and seabird bycatch estimates (in number) by target species for all 11 fishing areas from 1992 to 2017 from model M7 fitted to data from 11 fishing zones. Abbreviations represent the following: MIX – mixed species, SWO – swordfish (*Xiphias gladius*), TUN – tuna (*Tunnus* spp.), SHX – pelagic sharks (various Selachimorpha), DOL – dolphinfish (*Coryphaena hippurus*). Error bars are standard deviations. Plot is made using R package ggplot2 (version 3.3.2, https://ggplot2.tidyverse.org) in statistical program R (version 3.6.3, http://www.R-project.org/).

The estimated mean total seabird bycatch across all observer coverage areas in the U.S. Atlantic pelagic longline fishery from 1992 to 2017 was 3,066 (coefficient of variation, CV = 17.41%). The highest annual bycatch estimate was for 1997, with 560 birds on average (CV = 34.79%; Fig. 6a). The bycatch estimate was higher in the fishing zones along the East Coast (i.e. NEC, MAB, SAB) and in the Gulf of Mexico (GOM) than in other regions (one-way analysis of variance, *p* < 0.05). The MAB produced the greatest estimated total seabird bycatch (1,405 birds on average, CV = 19.91%), followed by NEC (742 birds on average, CV = 26.40%), SAB (312 birds on average, CV = 33.71%) and GOM (308 birds on average, CV = 33.51%; Fig. 6b). The highest seasonal estimate occurred in summer (1,271 birds on average, CV = 21.77%; Fig. 6c). Longline sets targeting the POP-designated mixed group of species were estimated to produce the highest seabird bycatch (1,855 birds on average, CV = 20.36%), followed by tuna (665 birds on average, CV = 24.76%) and swordfish (362 birds on average, CV = 29.95%; Fig. 6d).

The spatiotemporal interactive model (T7) fitted to data from the three East Coast high-bycatch zones produced higher bycatch estimates for these zones than were estimated for these zones by the constant spatial model (M7) fitted to data from all observer coverage areas (MAB, 2,324 birds on average, CV = 19.42%; NEC, 1,383 birds on average, CV = 25.36%; SAB, 750 birds on average, CV = 32.05%; Fig. 6b).

### Bycatch mitigation

**Figure 7:**
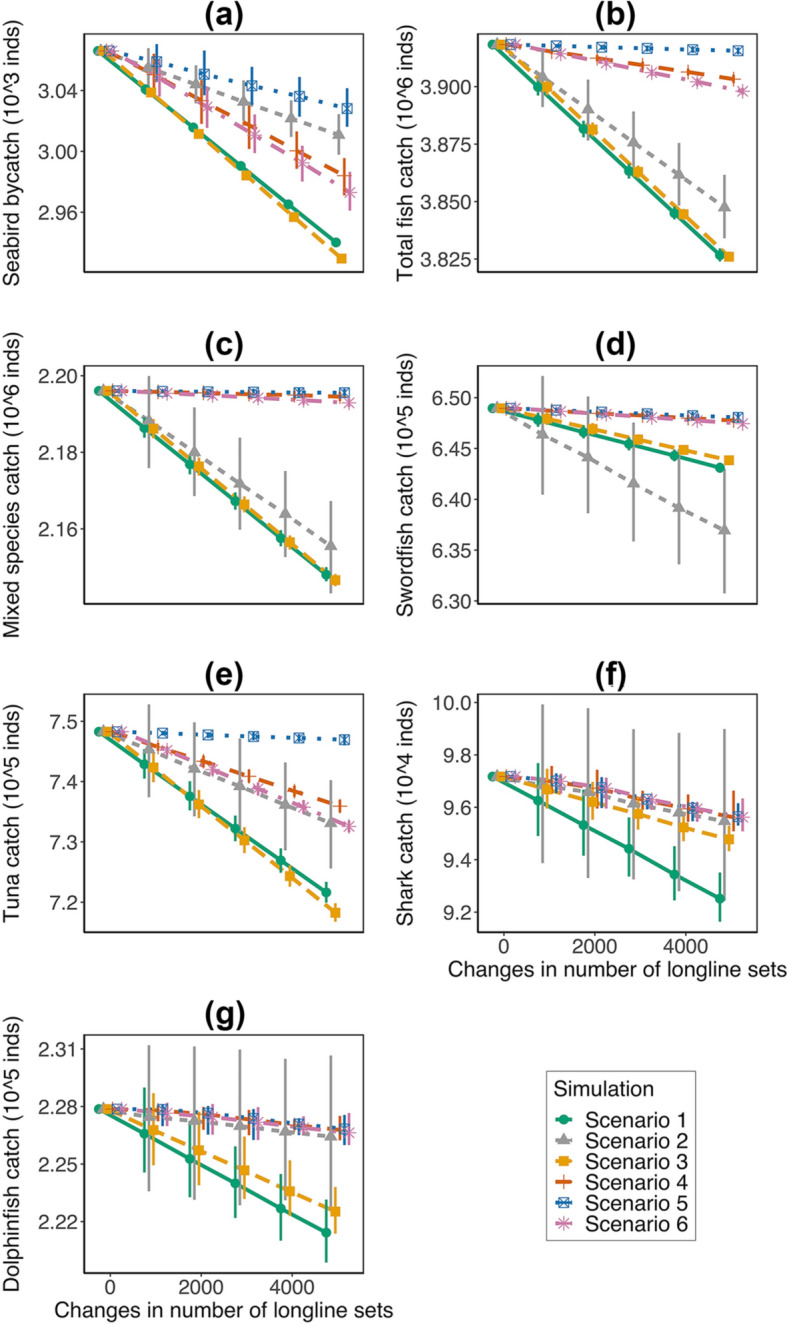
(**a**) Total number of bycatch estimates (individual birds) from 1992 to 2017, (**b**) total number of fish caught estimates (individual fish), (**c**) number of mixed species caught estimates (individual fish), (**d**) number of swordfish caught estimates (individual fish), (**e**) number of tuna caught estimates (individual fish), (**f**) number of pelagic sharks caught estimates (individual fish), (**g**) number of dolphinfish caught estimates (individual fish) under six scenarios of simulated change in fleet behavior. Point represents the mean value of the 1000 posterior means. Error bar represents the 95% credible interval. Plot is made using R package ggplot2 (version 3.3.2, https://ggplot2.tidyverse.org) in statistical program R (version 3.6.3, http://www.R-project.org/).

Simulated changes in fleet behavior proved able to decrease estimated seabird bycatch in the longline fishery (Fig. 7). Results based on removal or redistribution of 5000 sets indicated that estimated seabird bycatch decreased the most with removals: Scenario 3 (removal from the hotspot areas from summer-through-winter season, 5.39% decrease), followed by Scenario 1 (removal from the hotspot areas, 4.97% decrease). Scenarios 6 and 4, simulating redistributions from summer–winter and hotspots to spring and sites neighboring hotspots (Scenario 6: 3.67% decrease) and from hotspots to neighboring sites (Scenario 4: 3.24% decrease) were also effective. Set removal from anywhere during the summer-spring season (Scenario 2) or redistribution from summer–winter to spring (Scenario 5) tended to decrease the seabird bycatch less, 2.18% and 1.50% decrease, respectively. Of the four most efficient scenarios, redistributing effort from summer–winter and hotspots to spring and neighboring locations (Scenario 6) and redistributing longline sets from the hotspot locations (Scenario 4) showed the least negative impacts on fish catches, 0.52% and 0.38% decrease, respectively; However, scenarios 6 and 4 showed worse impacts on tuna catch than scenario 5 (Fig. 7e), potentially due to aggregation of tuna catch along the shelf break in the MAB and NEC, where the hotspots of seabird bycatch are located (Fig. S1 in the Supplementary information). For perspective, 5000 sets, affecting from 0.38% to 5.39% of seabird bycatch by their redistribution or removal, is about 1.83% of sets in the entire 1992–2017 database.

## Discussion

### Impacts of explanatory variables

Our analyses captured the significant impacts of year, season, target species, water temperature, set time and vessel ID on the probability of catching a seabird and the impact of number of hooks and haul time on positive seabird bycatch, and our results are consistent with previous studies in the same region^13,39^. The year effect on logit(*p*) decreases after 2004, when the circle hook replaced the J-hook (Fig. S2a in the Supplementary information). Another potential reason for the decrease is the decrease of fleets of trawlers for silver hake, Atlantic cod and other groundfish in the Northwest Atlantic, which resulted in a reduced prey base for seabirds that scavenge for these species at trawlers off the U.S. east coast, leading to a decrease in seabird abundance^40,41^, and a following decrease in seabird bycatch rate^9^. Most seabird longline bycatch species, such as herring gulls (*L. argentatus*) and great shearwaters (*Ardenna gravis*), return to their breeding colonies in spring^42,43^, and are less likely to be caught along the U.S. east coast. Most seabirds are visual feeders and forage during daytime, so setting and hauling longlines at night could limit birds’ assess to baited hooks^16^. The longline sets targeting dolphinfish produced the highest probability of catching a seabird, while longline sets targeting swordfish produced the lowest probability. In the POP data, longline sets targeting dolphinfish occurred in shallower waters (Fig. S2j in the Supplementary information), which increased seabird access to baited hooks^16^, and most longline sets targeting dolphinfish (91.17%) were hauled back during daytime (Fig. S2o in the Supplementary information) and all sets were not weighted (Fig. S2p in the Supplementary information), potentially increasing the chance to catch a seabird^16,43^. About 40% of longline sets targeting swordfish were set at night (Fig. S2m in the Supplementary information), when seabird bycatch is less likely. The influence of water temperature might have been through the copepod-sand lance-seabird food chain^45^. Increased availability of *Calanus finmarchicus* at colder temperatures may attract more seabirds, increasing the overlap with fisheries and thus increasing bycatch risk.

The vessel ID could impact seabird bycatch probability, in accordance with previous studies^46^. The “vessel 38”, “vessel 146” and “vessel 167” have greater posterior mean effect (Fig. 3a). The “vessel 38” caught seabirds on 8 longline sets, and all of these 8 sets occurred in the MAB; this vessel primarily fished in the MAB (149 sets), but also fished in the SAB (55 sets), NEC (24 sets), Florida east coast (FEC, 27 sets), GOM (9 sets) and Caribbean region (CAR, 1 set). The “vessel 146” caught seabirds on 9 longline sets, among which 8 sets occurred in the MAB and 1 set occurred in the NEC; this vessel primarily fished in the MAB (125 sets), but also fished in the SAB (31 sets) and NEC (6 sets). The “vessel 167” caught seabirds on 3 longline sets, and all 3 of these sets occurred in the NEC; this vessel fished mainly in the TUN (71 sets), but also fished in the NEC (14 sets) and MAB (9 sets). By including the vessel effect in the model, we removed its influence (the vessel ID artifact) on the spatial field.

The impacts of factors such as hook depth, hook type and additional weight were not significant, possibly because effects of these factors were confounded by their correlations with other factors such as year and target species (Fig. S2 in the Supplementary information). Although other factors, such as fish discard, have been found related to seabird bycatch in other studies^10^, their impacts are not significant in the present study. Seabirds may be attracted by fishing vessels since fisheries discard is a predictable and abundant and readily accessible source of food^47,48^. However, intense fishing activities may cause depletion of fish stocks with a reduction of natural prey available for seabirds, and fisheries discards are less nutritious compared with natural prey of seabirds^49^. Few analyses of field data have shown the importance of fishing activities on seabird aggregation in comparison with the distribution of marine habitats and the availability of natural food sources^50^.

### Spatial pattern of seabird bycatch

The spatial effect introduced into this study is particularly appropriate for seabirds because of their migratory behavior and wide distribution. The simulated spatial field in the model captured the spatial heterogeneity of seabird bycatch events not explained by other influential variables (e.g., year, season, water temperature, and fishery-related variables such as target species, hook type, bait type, additional weight, vessel).

The highest seabird bycatch probability occurs in the MAB, NEC and SAB areas, likely associated with high seabird density, activity and diversity in these areas. The MAB, characterized by its high freshwater inflow, expansive hard bottom, and meeting of warm Gulf Stream and cold Labrador Current waters^51,52^, appears to be especially favorable habitat for pelagic fishes, attracting both more seabirds and more intensive pelagic longline fishing. The occurrence of at least 49 seabird species has been documented at the outer continental shelf off Cape Hatteras near the boundary of the SAB with the MAB^53^.

In the three East Coast high-bycatch zones, the hotspots vary in presence and location year by year, and the changes link to the position of the Gulf Stream. After separating from the U.S. coast, the Gulf Stream is prone to meander and is frequently accompanied by long-lived mesoscale eddies. The warm-core eddies or rings off a northward bending meander have been found to diminish primary productivity locally, bringing tropical species to the continental shelf where colder slope water is found. The life cycle of a warm core ring is generally a few months to a year. The cold-core eddies or rings off a southward bending meander can persist 1 to 4 years and are found to the south of the Stream, where nutrient-rich waters can upwell from deeper waters, supporting high primary productivity and partly controlling foraging behavior and displacement of marine top predators, including large fishes, birds, turtles and marine mammals^54^, and then influence the distribution of pelagic longline fishing. Seabirds could track these mesoscale eddies to locate food patches^54^, and the increased overlap with fisheries in the northern area would increase bycatch risk. A year with a greater GSNW index is one in which the Gulf Stream meanders follow a more northerly track. The track influences foraging behavior of seabirds, possibly with a time lag through food chain transfer^39^.

### Seabird bycatch estimates in the U.S. Atlantic pelagic longline fishery

Our estimates of seabird bycatch in the U.S. Atlantic pelagic longline fishery show clear spatiotemporal patterns (Fig. 6), corresponding to previous studies^13,22^. The spatiotemporal variation of estimated seabird bycatch does not correspond with variation in U.S. Atlantic pelagic longline fishing effort. For example, 35% of the total number of pelagic longline sets occurred in the GOM, twice the number of longlines sets in the MAB, but the seabird bycatch estimate in the MAB was about four times the GOM estimate (Fig. 6b). Longline sets were fewest in winter, but the seabird bycatch estimate for spring was less than half that of winter and even lower in comparison to the bycatch of summer and fall (Fig. 6c). Spatiotemporal variation in seabird distribution plays a significant role in explaining the observed spatiotemporal pattern of seabird bycatch estimates.

The highest annual seabird bycatch estimate occurred in 1997, corresponding to the highest seabird catch rate in the POP data. In the POP data, a total of 11 longline sets captured 33 seabirds in 1997. All 33 seabirds were captured in summer, and 21 were captured in the NEC. The number of pelagic longline sets in the NEC in summer were also highest in 1997, which might have contributed to the high estimate of seabird bycatch. Some birds frequently captured in the POP, like herring gulls and great shearwaters (Table S1 in the Supplementary information), return to their breeding colonies when it is springtime off the U.S. coast, which may explain the relatively low spring bycatch by the fishery^42,43^. The longline sets targeting mixed fish species produced the highest seabird bycatch estimate (Fig. 6d). In the POP data, 50 of the 92 longline sets with seabirds targeted mixed fish species. Of these 50 longline sets, 72% occurred before the J-hook was prohibited beginning in August 2004. The J-hook has been found to be associated with higher seabird bycatch probability^55^. The proportion of pelagic longline sets targeting mixed species was also highest (50%), contributing to the higher seabird bycatch estimate from sets targeting mixed species.

### Spatiotemporal interaction

The spatiotemporal interaction was expected to improve model performance. However, when fitted to data from the 11 fishing zones as a whole, a spatiotemporal interactive model did not show its superiority over the constant spatial model (M7; Table 1). The seabird bycatch events were too sparse in the study region overall (Fig. 1) to support a complex spatiotemporal model, which needs better spatiotemporal representation in the data. In contrast, the spatiotemporal interactive model (T7) was the best-performing model when fitted to data confined to the three East Coast high-bycatch zones. The lower bycatch estimates from M7 for the three high-bycatch zones (Fig. 6b) reflected bias caused by low bycatch rates in neighboring zones; the low bycatch rates in other zones tended to buffer the bycatch estimates in the three high-bycatch zones. These results suggest that, where seabird bycatch is concentrated, as off the U.S. east coast, the spatiotemporal interactive model may be appropriate to estimate seabird bycatch; the constant spatial model may be the better choice where data are too sparse to be informative, as in the longline fishing area as a whole.

The lower CVs of estimates from model T7 for the three high-bycatch zones supports the choice of the spatiotemporal interactive model in zones with high bycatch. Previous models, including other types of spatial models, applied to earlier versions of POP longline data, have resulted in higher rather than lower CVs when applied to the three high-bycatch zones in comparison to corollary estimates for these three zones from models of the full study area^13^. Since CV is a criterion of quality in total fleet bycatch estimation (with a target of 30% or less based on the recommendation of the National Working Group on Bycatch), the reduction in CV in the high bycatch areas might be considered another advantage of this new approach.

### Implication for seabird bycatch mitigation

Standard deck practices such as bird-scaring lines, weighted lines, blue-dyed baits and night setting have been proved to efficiently mitigate seabird bycatch in longline fisheries^14–16^. In the POP, several mitigation measures, such as circle hook, weighted lines, night setting, were taken to decrease bycatch of other species like turtles, but not for seabirds. We examined the effects of these mitigation measures on seabird bycatch in this study and found that some mitigation measures could also decrease seabird bycatch, for example, night setting and hauling produced less seabird bycatch. As an alternative to immediate management action, a spatial bycatch avoidance would be helpful. However, the impact and spatial distribution of bycatch is frequently unknown making it difficult to develop effective mitigation strategies. This study, built on a previous study on hotspot analysis, was intended to investigate spatial relocation as a bycatch mitigation strategy by evaluating the percent of bycatch decrease and the percent of harvest being influenced.

Understanding and predicting spatiotemporal changes in seabird bycatch from fisheries might provide a means to mitigate seabird bycatch in addition to standard deck practices. In the present study, seabird bycatch estimates were significantly higher along the East Coast of the U.S. (i.e. MAB, NEC, and SAB areas) and during summer through winter. The inter-annual variations in bycatch hotspots are linked to the meridional position of the Gulf Stream, which is likely correlated with the NAO and the Gulf Stream position in the past^56^. Our previous study found that a higher NAO index, which corresponds to stronger westerly and trade winds, favored more northerly paths of the Stream about two years later^56^. The time-delay might be associated with the adjustment time of the ocean circulation^56^. A tendency for displacements of the north wall to persist for more than a year was also found^56^. Thus, a large part of interannual variation in the Gulf Stream position can be predicted using the NAO index two years previously and the previous year’s Gulf Stream position^56^.

Our simulation results demonstrate that an appropriate combined regional and seasonal readjustment of fishing effort, such as fishing effort shifted away from bycatch hotspots and seasons with high seabird bycatch to neighboring sites and seasons with low bycatch, could significantly reduce seabird bycatch in the U.S. Atlantic pelagic longline fishery while largely maintaining fishers’ benefit. For the tuna fishery, a more directional redistribution is required to better maintain fishers’ benefit; for example, moving fishing fleets northward or southward along the shelf break, but away from the hotspots of seabird bycatch shown in Fig. 5. Our simulation results depended on the assumption that relocating fishing effort would not change the spatial distribution of seabird bycatch. One might think that because birds follow boats for feeding, moving fleet effort would change seabird spatial distribution; however, the influence of the possible attraction of birds to boats on outcomes of our proposed mitigation strategy depends upon geographic scale. Seabird attraction to boats is likely strongest at the local scale; (Skov and Durinck in 2001 found that seabird attraction to fishing vessels is a local process, less than 10 km in the Baltic Sea-North Sea gradient^50^), whereas effort was moved at least 50 miles away in our experiments. Furthermore, we have substantial evidence that the attraction of birds to fishing boats is not a significant influence on bird patterns in relation to vessel patterns from our analyses of the longline POP and logbook data bases. First, discard did not significantly affect seabird bycatch rate in the present study, indicating a weak interaction between seabirds and fishing vessels. Second, performance of our hurdle model of seabird bycatch was not improved by including fish catch. Third, estimated seabird bycatch did not have the same pattern of spatial distribution as logbook-noted target fish species caught (Fig. S1 in Supplementary information). Clearly, birds did not simply aggregate in areas with intense fishing activity or higher fish catches. Nevertheless, finer detailed knowledge of the spatial distribution of seabird abundance and its relationship with fishing activity in the western North Atlantic pelagic longline fishery likely would enhance seabird bycatch hotspot analysis and the information available to mitigate seabird bycatch by redirecting fishing activity.

Strategies to reduce seabird bycatch in the western North Atlantic pelagic longline fishery should include implementing real-time seabird bycatch hotspot avoidance by fishing industry vessels furnished with model predictions based on long-term climate oscillations^57^, or increasing fleet communication to enable vessels to coordinate avoidance of areas and/or time periods when seabirds aggregate^58,59^. A voluntary bycatch avoidance program, relying on consistent communication, was proved successful to decrease bycatch of river herring and American shad in the northwest Atlantic mid-water trawl fishery targeting Atlantic herring and Atlantic mackerel^59^. Increasing fisheries monitoring frequency and coverage, especially along the U.S. East Coast during summer through winter, should provide more detailed fine-scale knowledge of fleet and seabird movement patterns and their influencing factors and improve the predictions to guide seabird bycatch mitigation through effort removal or redistribution.

## Methods

### POP and logbook data

Pelagic longline fishery data and seabird bycatch observer data were obtained from the National Marine Fisheries Service (NMFS) Logbook Program and POP, respectively. In the POP, vessels were randomly selected based on their fishing effort and location for each POP statistical area in the previous year. Observers were placed in rotation based on when they last returned from a trip and debriefed while accounting for any time off requests. The POP observer data recorded 19,811 longline sets during 1992 to 2017 in the 11 fishing zones, ranging between 287 and 1,483 annually. Ninety-two of the observer sets had positive seabird bycatch for a total of 165 birds. More than 99% of sets had zero seabird bycatch. More than 88% of the total observed longline sets were deployed in the GOM (9,020 sets), MAB (3,053 sets), SAB (2,181 sets), FEC (2,052 sets) and NEC (1,235 sets) areas. Most seabirds (86 birds) were caught in the MAB, followed by 44 birds in the NEC and 19 birds in the SAB. The POP longline sets in spring (6,626 sets) and summer (5,403 sets) were more than in fall (4,244 sets) and winter (3,538 sets). Among the 165 seabirds caught, 55 birds were unspecified, most of which were captured prior to 2004, before seabird identification training was provided as a part of the POP training program. Grouped by their minimal identifications, gulls (*Larus* sp.) were the most frequently captured (54 birds), followed by shearwaters (*Procellariidae* spp., 33 birds; especially great shearwaters, *Ardenna gravis*, 27 birds), and northern gannets (*Morus bassanus*, 17 birds) (Table S1).

A total of 273,002 logbook sets out of the 285,589 sets that we examined had sufficient information to match a refined set of POP data. Among the 273,002 logbook sets, 87% of the total pelagic longline sets occurred in the GOM (95,285 sets), MAB (48,176 sets), FEC (39,127 sets), SAB (33,794 sets) and NEC (20,304 sets) areas. Number of longline sets has declined almost steadily since 1995, the total number of hooks in each year also peaked in 1995; whereas the number of hooks per set increased steadily until 2005. During the entire period, number of sets deployed was greatest in summer (84,972 sets) and least in winter (53,829 sets). Most longline sets targeted mixed fish (usually a mix of swordfish and tuna) species (135,853 sets), followed by swordfish (66,726 sets) and tuna (64,316 sets), and fewer targeted pelagic sharks (3,544 sets) and dolphinfish (2,563 sets).

Variables recorded in the POP data are listed in Table 3. The commonly used hook types included 16/0 and 18/0 circle hooks, and 8/0 and 9/0 J-hooks. Prior to August 2004, both the circle hook and the J-hook were used in U.S longline fisheries. Starting in August 2004, the use of the circle hook was mandated to reduce sea turtle bycatch (69 Fed. Reg. 40,734). Only those variables that were found to significantly impact seabird bycatch rate and were recorded in the logbook data were used to extrapolate from the observed bycatch to total estimated bycatch.

**Table 3:** Potential explanatory variables considered in this study. The real vessel ID was recoded as numbers because of the confidential concerns.

Variables	Type	Categories/mean	Units
Vessel ID	Categorical	Vessel 1, vessel 2, vessel 3, etc. (274 vessels in total)	
Observer ID	Categorical	2, 3, 4, 5, etc. (180 observers in total)	
Year	Categorical	1992–2017	
Season	Categorical	Winter, Spring, Summer, Fall	
Target species	Categorical	Mixed species, Swordfish, Tuna, Shark, Dolphinfish	
Longitude	Continuous	− 79.39	°W
Latitude	Continuous	30.47	°N
Water temperature	Continuous	24.98	°C
Water depth	Continuous	1122.00	m
Wind speed	Continuous	11.78	kn
Wind direction	Continuous	150.20	◦
Wave height	Continuous	3.41	ft
Hook type	Categorical	Circle hook (10/0,13/0, 15/0,16/0, 18/0, 20/0), J-hook (7/0, 8/0, 9/0, 10/0, 11/0, 13/0, 14/0, 15/0)	
Mainline length	Continuous	30.10	mi
Number of hooks	Continuous	717.10	
Set speed	Continuous	7.08	kn
Hook depth	Continuous	27.75	m
Additional weight	Continuous	4.47	lb
Set duration	Continuous	3.70	hr
Haul duration	Continuous	6.22	hr
Soak duration	Continuous	8.36	hr
Bait type	Categorical	Mackerel, Squid	
Set time	Categorical	Day (6:30–19:30 spring, 5:30–19:30 summer, 7:00–18:30 fall, 7:30–18:00 winter), Night	
Haul time	Categorical	Day (same as set time), Night	
Discard	Continuous	15.88	# of fish discarded/set
Fish catch	Continuous	15.29	# of fish kept/set

### Model framework

A set of hurdle models with a probability component and a positive-bycatch component were fitted to model seabird bycatch. The product of estimates from these two components gave the expected seabird bycatch (i.e., number caught) by a longline set.

The probability sub-model assumed that the event of capturing or not-capturing a seabird in a longline set followed a binomial distribution with a log link:

$$ {\text{logit}}(p) = {\text{intercept}} + \sum {f_{i} (c_{i} )} + \sum {s_{j} (x_{j} )} + \xi_{z} $$

where *p* is the probability of catching a seabird; *c*_*i*_ represents the *i*th categorical variables; *x*_*j*_ is the *j*th continuous variable; *s* is a smoothing function, defined through a first-order random walk (RW1) process^29^ (for details, please see Supplementary information); $\xi_{z}$ represents the spatial effect, and, in spatiotemporal interactive models, $\xi_{z}$ is extended to $\xi_{z}^{t}$ to capture time-varying spatial heterogeneity.

The positive-bycatch sub-model assumed that the positive number of seabirds caught in a longline set followed a zero-truncated Poisson distribution with a log link, and the mean was:

$$ \begin{aligned} & \log ({\text{positive number of seabird bycatch per longline set}}) = {\text{intercept}} + \sum f_{i} \left( {c_{i} } \right) \\ & \quad + \sum s_{j} \left( {x_{j} } \right) + \xi_{y} \\ \end{aligned} $$

### Model fitting and comparison

**Figure 8:**
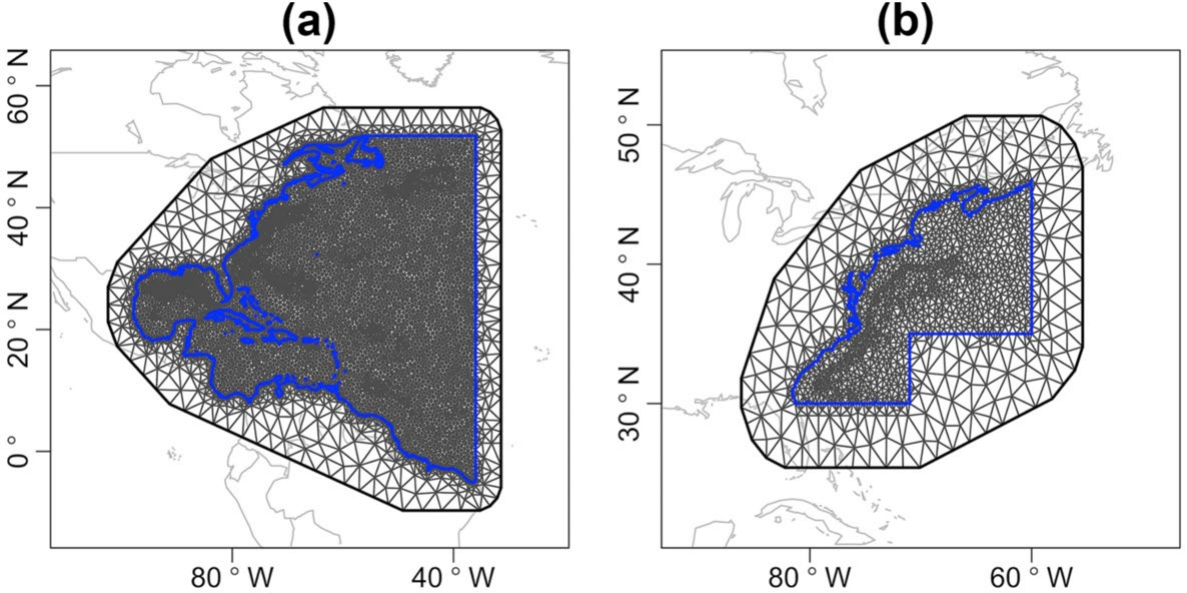
(**a**) Mesh for all observer coverage area (11 fishing zones). (**b**) Mesh for the three high-bycatch zones. The blue line is the domain boundary. The SPDE edge effect is moved outside the domain of interest using an extension with larger triangles. Plot is made in statistical program R (version 3.6.3, http://www.R-project.org/). Map data is from R package maps (version 3.3.0, https://CRAN.R-project.org/package=maps).

The first step of developing the model was to construct a triangular mesh over the study region (Fig. 8), and then the POP data were projected onto the mesh. Sparse basis functions were evaluated over adjacent mesh nodes and used to approximate the spatial effect^32^. The mesh was extended a bit outside the region of interest to reduce boundary effects such as larger variance at the boundary^60^.

Spatial random effects ($\xi_{z}$, $\xi_{z}^{t}$, $\xi_{y}$ and $\xi_{y}^{t}$) were modeled by Gaussian Random Fields (GRF)^27^. A GRF is specified through its mean function and covariance function, e.g. Matérn covariance function. In a stationary spatial model, the covariance function between two points depends on their distance^32^. In this study, the covariance function was interpreted as a collection of paths between two points through a Simultaneous Autoregressive (SAR) model^33^. The model used, a Barrier model, reduced bias caused by coastlines by cutting off the paths that crossed the coastlines^33^.

GRFs are hampered by a high computational cost, known as “the big *n* problem”^27^. The SPDE approach overcomes the problem through representing a GRF with a Matérn covariance function with a discretely indexed Gaussian Markov Random Field (GMRF) within the INLA framework^29,32^. The solution to the SPDE is a Matérn field that is a special case of a GRF^32^. A GMRF representation is achieved by approximating the solution using a piecewise linear basis function representation defined in the triangulation of the domain of interest^32^. All analyses were performed using R^61^ and the R-INLA package^62^. The default and recommended settings for priors were adopted^31,33,60,63,64^ (for details please see Supplementary information).

We ran a forwards stepwise variable selection process, in which a step involved testing the addition of each covariate separately. We started with a base model only incorporating the intercept. The spatial effect ($\xi$) was incorporated in the final step. Support for models with different explanatory variables was compared based on the deviance information criterion (DIC)^36^ and Watanabe-Akaike information criterion (WAIC)^37^. The DIC is defined as:

$$ {\text{DIC}} = \overline{D} + p_{D} $$

where $\overline{D}$ is the posterior mean of the deviance of the model, and *p*_*D*_ is the effective number of parameters in the model^33^.

The WAIC is defined as:

$$ {\text{WAIC}} = - 2*(LPPD - p_{D} ) $$

where *LPPD* is the log posterior predictive density^37^. It is recognized that the DIC may under-penalize and select over-parameterized models over simpler models^65^. It is also known that the DIC can produce negative estimates of the effective number of parameters in a model. The WAIC is fully Bayesian and uses the entire posterior distribution, so it is recommended over the DIC criterion^37,66^. The WAIC was computed to validate the DIC in this study. Alternative models with smaller DIC and WAIC values perform better^66^. A reduction of 5 in the DIC or WAIC indicates a significantly better prediction quality of the model^66^.

### Bycatch estimate

Barrier hurdle models were developed using POP data and then applied to fishery logbook data to obtain an estimate of seabird bycatch for each longline set. We generated 1000 samples from the approximated posterior distribution for each longline set. A bycatch estimate was computed for each of these 1000 posterior samples, and a mean bycatch estimate was obtained for each longline set. The CV of the bycatch estimate was estimated to reflect its uncertainty.

### Simulations

Six simulations of change in fleet effort provided the opportunity to compare the effect of redistributing longline sets to simply removing them. Scenarios 1, 2 and 3 simulated the effects of set removal from the hotspot areas (Scenario 1), from anywhere during the summer-spring season (Scenario 2), or the hotspot areas from summer-through-winter season (Scenario 3). Scenarios 4, 5, and 6 simulated the effect of redistributing sets from hotspots to neighboring sites (Scenario 4), from summer–winter to spring (Scenario 5), or from summer–winter and hotspots to spring and locations away from hotspots (Scenario 6). Sites to which effort was redistributed were at least 50 miles from hotspots. Each scenario was repeated 1000 times to yield 1000 sets of results. The corresponding changes in bycatch estimates were compared to gage the effectiveness of the various strategies, both in reducing seabird bycatch and minimizing impact of the changes on total fish catch, a rough index of fishers’ benefit (for how to predict fish catch for redistributed longline sets please see Supplementary information).

## Disclaimer

The scientific results and conclusions, as well as any views or opinions expressed herein, are those of the author(s) and do not necessarily reflect those of NOAA or the Department of Commerce.

## Supplementary Information

Supplementary Information.
