# Supplementary material for: Climate driven spatiotemporal variations in seabird bycatch hotspots and implications for seabird bycatch mitigation
Source: Sci Rep. 2021 Oct 19;11:20704. doi: 10.1038/s41598-021-00078-z (PMC8526677; doi:10.1038/s41598-021-00078-z)
Supplement: Supplementary file 1 — Supplementary Information. [file 41598_2021_78_MOESM1_ESM.docx]

**Title:** Climate driven spatiotemporal variations in seabird bycatch hotspots and implications for seabird bycatch mitigation

Authors: Rujia Bi*, Yan Jiao, Joan A. Browder

Rujia Bi. [rbi@vt.edu](mailto:rbi@vt.edu). Department of Fish and Wildlife Conservation, Virginia Polytechnic Institute and State University, Blacksburg, VA 24061, USA.

Yan Jiao. [yjiao@vt.edu](mailto:yjiao@vt.edu). Department of Fish and Wildlife Conservation, Virginia Polytechnic Institute and State University, Blacksburg, VA 24061, USA.

**Joan A. Browder**. [Joan.Browder@noaa.gov](mailto:Joan.Browder@noaa.gov). NOAA National Marine Fisheries Service, Southeast Fisheries Science Center, Miami, FL 33149, USA.

*Corresponding author: Rujia Bi. Department of Fish and Wildlife Conservation, Virginia Tech, Blacksburg, VA 24061, USA. Tel: (409) 692 - 1794. Email: [rbi@vt.edu](mailto:rbi@vt.edu).

**Supplementary Information**

**Model details**

The first-order random walk (RW1) process model assumes that *f*($t_{i}$) - *f*($t_{i-1}$) ~ *N*(0, $\tau^{-1}$); where $\tau$ is a precision parameter. The RW1 process is constrained to sum to zero, and rescaled to have typical variance 1 when $\tau$ = 1.

We assigned a flat non-informative prior for the intercept ~ *N*(0, $\infty$), a log-gamma prior on the log-precision (*a* = 0.1, *b* = 0.1) for the effects of year, season, target species and set time. For the smooth functions, we assumed a Penalized Complexity (PC) prior on the log-precision ($u$ = 0.5, $\alpha$ = 0.01). For the spatial effect of all observer coverage area, we assigned a joint PC prior on range ($\rho$) and standard deviation ($\sigma$): prior.range = c(25, 0.5) specifying that there is an priori probability of 50% that the range will be less than 25, and prior.sigma = c(3, 0.01) specifying that there is an priori probability of 1% that the standard deviation will be larger than 3. For the spatial effect of the three high-bycatch zones, we assigned a joint PC prior on range ($\rho$) and standard deviation ($\sigma$): prior.range = c(6, 0.5) specifying that there is an priori probability of 50% that the range will be less than 6, and prior.sigma = c(3, 0.01) specifying that there is an priori probability of 1% that the standard deviation will be larger than 3.

**Predict fish catch for redistributed longline sets**

The number of a fish species caught in a longline set recorded in the logbook data was fitted using a Poisson distribution with a log link, and the mean was:

log(number of fish caught per longline set) = intercept + + *f*(year) + *f*(season) + *s*(water temperature) + *f*(set time) + *s*(soak duration) + *s*(number of hooks) + $\xi$

where $\xi$ represents spatial effect. In simulation experiments, fish catches were predicted for redistributed longline sets through the model.

**Table S1.** Number of seabirds caught in the POP by area. Abbreviations represent the following: NED – Northeast district, NCA – North Central Atlantic, TUN – Tuna North, TUS – Tuna South, NEC – Northeast coast, SAR – Sargasso region, CAR – Caribbean region, MAB – Mid-Atlantic bight, SAB – South Atlantic bight, FEC – Florida east coast, GOM – Gulf of Mexico.

| **Family** | **Species** | **NED** | **NCA** | **TUN** | **TUS** | **NEC** | **SAR** | **CAR** | **MAB** | **SAB** | **FEC** | **GOM** | **Total** |  |
| --- | --- | --- | --- | --- | --- | --- | --- | --- | --- | --- | --- | --- | --- | --- |
| Laridae | Herring gull (*Larus argentatus*) | 0 | 0 | 0 | 0 | 3 | 0 | 0 | 13 | 1 | 0 | 1 | 18 |  |
|  | Laughing gull (*Larus atricilla*) | 0 | 0 | 0 | 0 | 0 | 0 | 0 | 0 | 1 | 0 | 2 | 3 |  |
|  | Black-backed gull (*Larus marinus*) | 0 | 0 | 0 | 0 | 0 | 0 | 0 | 10 | 0 | 0 | 0 | 10 |  |
|  | Other Laridae spp. | 0 | 0 | 0 | 0 | 2 | 0 | 0 | 21 | 0 | 0 | 0 | 23 |  |
| Procellariidae | Great shearwater (*Ardenna gravis*) | 1 | 0 | 0 | 0 | 7 | 0 | 0 | 18 | 0 | 0 | 1 | 27 |  |
|  | Cory's shearwater  (*Calonectris diomedea*) | 0 | 0 | 0 | 0 | 0 | 0 | 0 | 1 | 1 | 0 | 0 | 2 |  |
|  | Northern fulmar (*Fulmarus glacialis*) | 1 | 0 | 0 | 0 | 0 | 0 | 0 | 1 | 0 | 0 | 0 | 2 |  |
|  | Other *Procellariidae* spp. | 0 | 0 | 1 | 0 | 1 | 0 | 0 | 2 | 0 | 0 | 0 | 4 |  |
| Sulidae | Northern gannet (*Morus bassanus*) | 1 | 0 | 0 | 0 | 3 | 0 | 0 | 8 | 4 | 0 | 1 | 17 |  |
| Pelecanidae | Brown pelican  (*Pelecanus occidentalis*) | 0 | 0 | 0 | 0 | 0 | 0 | 0 | 0 | 0 | 0 | 3 | 3 |  |
| Oceanitidae | Wilson's storm petrel  (*Oceanites oceanicus*) | 0 | 0 | 0 | 0 | 0 | 0 | 0 | 1 | 0 | 0 | 0 | 1 |  |
| Stercorariidae | Arctic skua  (*Stercorarius parasiticus*) | 0 | 0 | 0 | 0 | 0 | 0 | 0 | 0 | 0 | 0 | 1 | 1 |  |
| Unidentified | Aves | 1 | 0 | 0 | 0 | 28 | 0 | 0 | 11 | 12 | 0 | 2 | 54 | |

**Figure legend**

**Fig. S1.** Posterior mean of the spatial random field on the number of log(number of fish caught per longline set) for each species when fitted to the logbook data. Abbreviations of target species are as follow: MIX – mix of swordfish and tuna species, SWO – swordfish, TUN – tuna, SHX – pelagic sharks, DOL – dolphinfish. Abbreviations of fishing zones are as follow: NED – Northeast district, NCA – North Central Atlantic, TUN – Tuna North, TUS – Tuna South, NEC – Northeast coast, SAR – Sargasso region, CAR – Caribbean region, MAB – Mid-Atlantic bight, SAB – South Atlantic bight, FEC – Florida east coast, GOM – Gulf of Mexico.

**Fig. S2.** (a) Compositions of hook types across years; (b) compositions of bait types across years; (c) distributions of hook depth across years; (d) compositions of target species across years; (e) compositions of day/night setting across years; (f) compositions of day/night hauling across years; (g) compositions of weighted or not across years; (h) compositions of fishing seasons across years; (i) compositions of fishing zones across years; (j) distributions of hook depth for target species; k) compositions of hook types for target species; (l) compositions of bait types for target species; (m) compositions of day/night setting for target species; (n) compositions of fishing zones for target species; (o) compositions of day/night hauling for target species; (p) compositions of weighted or not for target species. Abbreviations of target species are as follow: MIX – mix of swordfish and tuna species, SWO – swordfish, TUN – tuna, SHX – pelagic sharks, DOL – dolphinfish. Abbreviations of fishing zones are as follow: NED – Northeast district, NCA – North Central Atlantic, TUN – Tuna North, TUS – Tuna South, NEC – Northeast coast, SAR – Sargasso region, CAR – Caribbean region, MAB – Mid-Atlantic bight, SAB – South Atlantic bight, FEC – Florida east coast, GOM – Gulf of Mexico.

**Fig. S3.** Histogram of mainline length in the POP data.

**
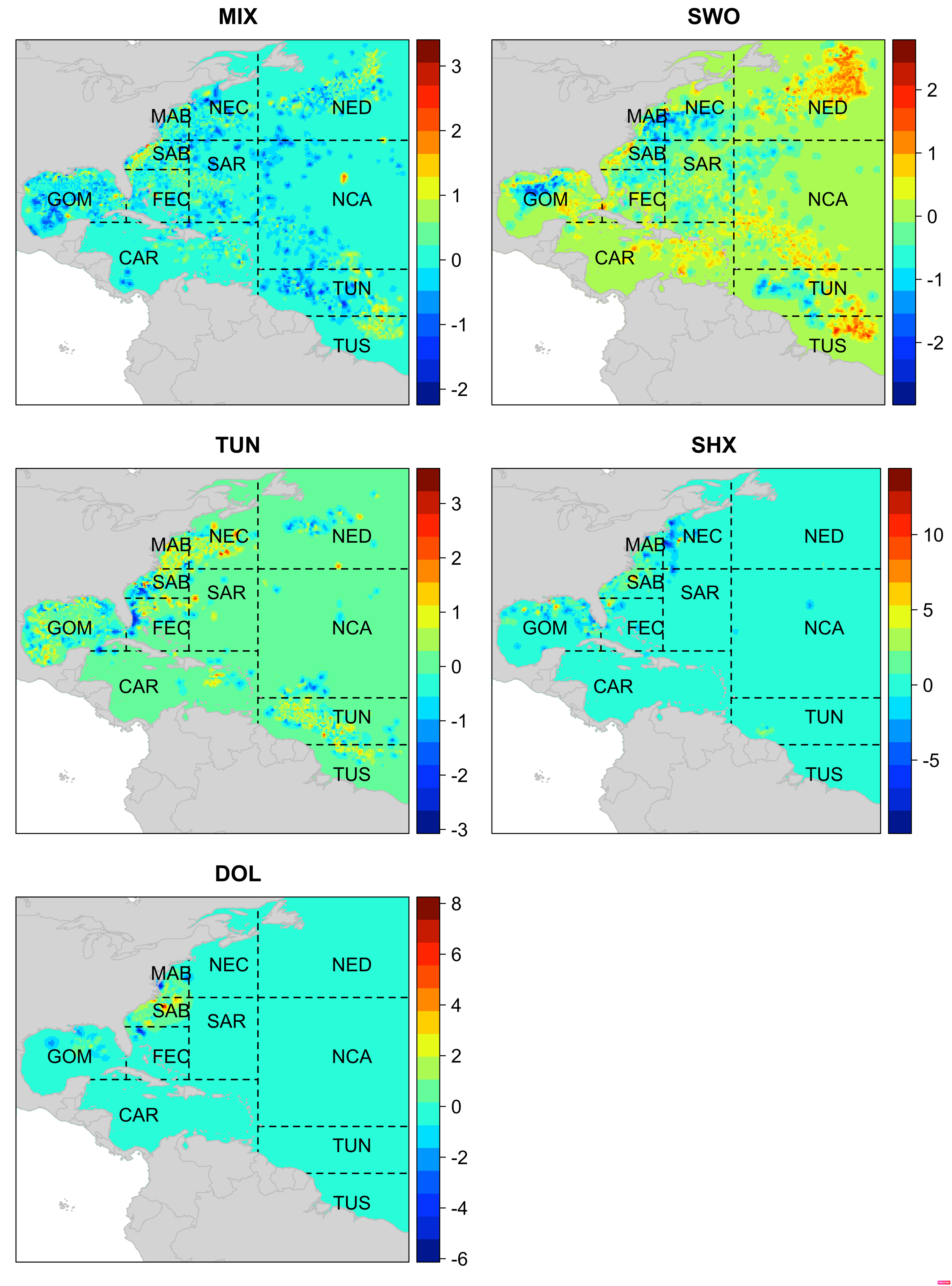
**

**Fig. S1**


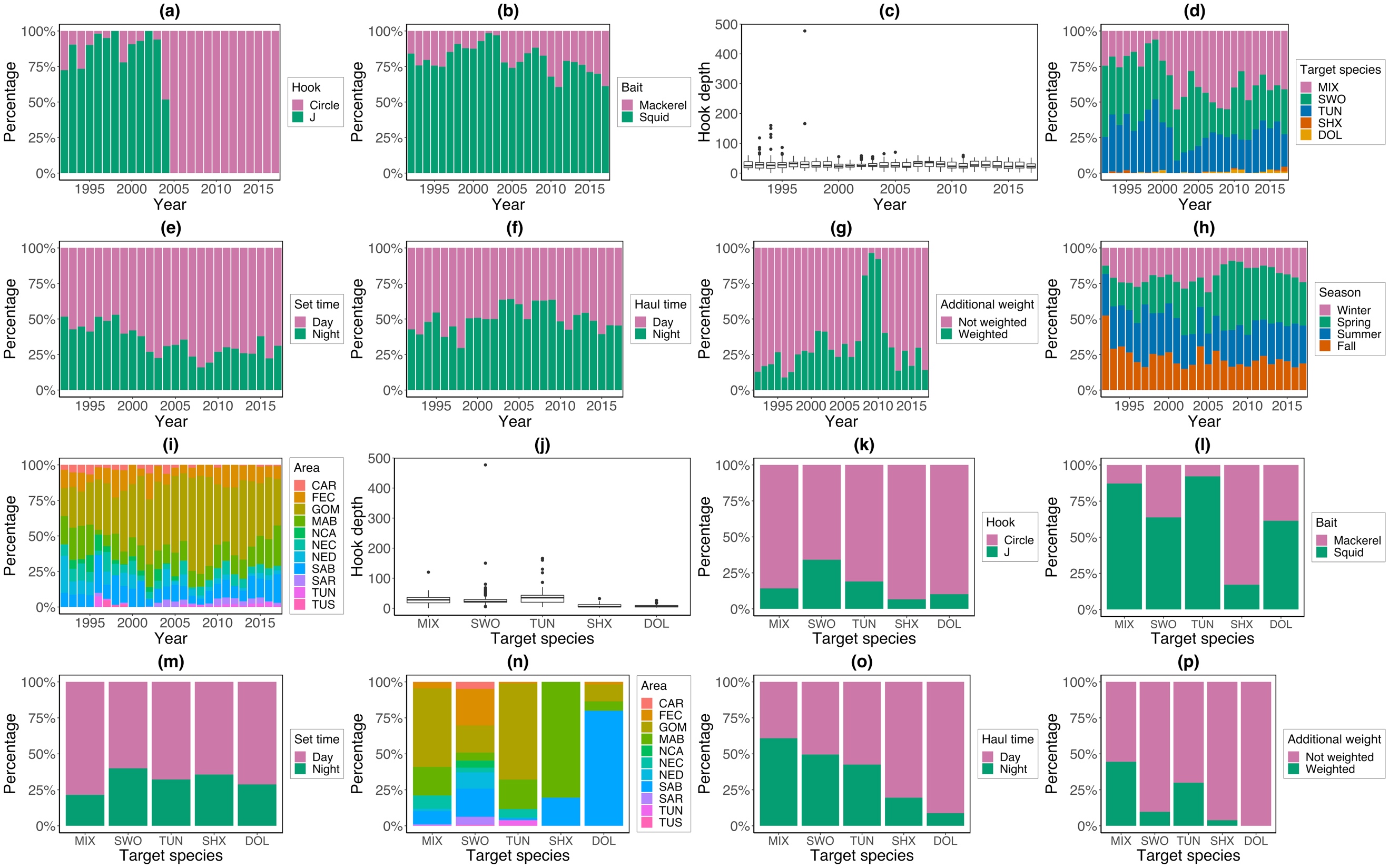


**Fig. S2**


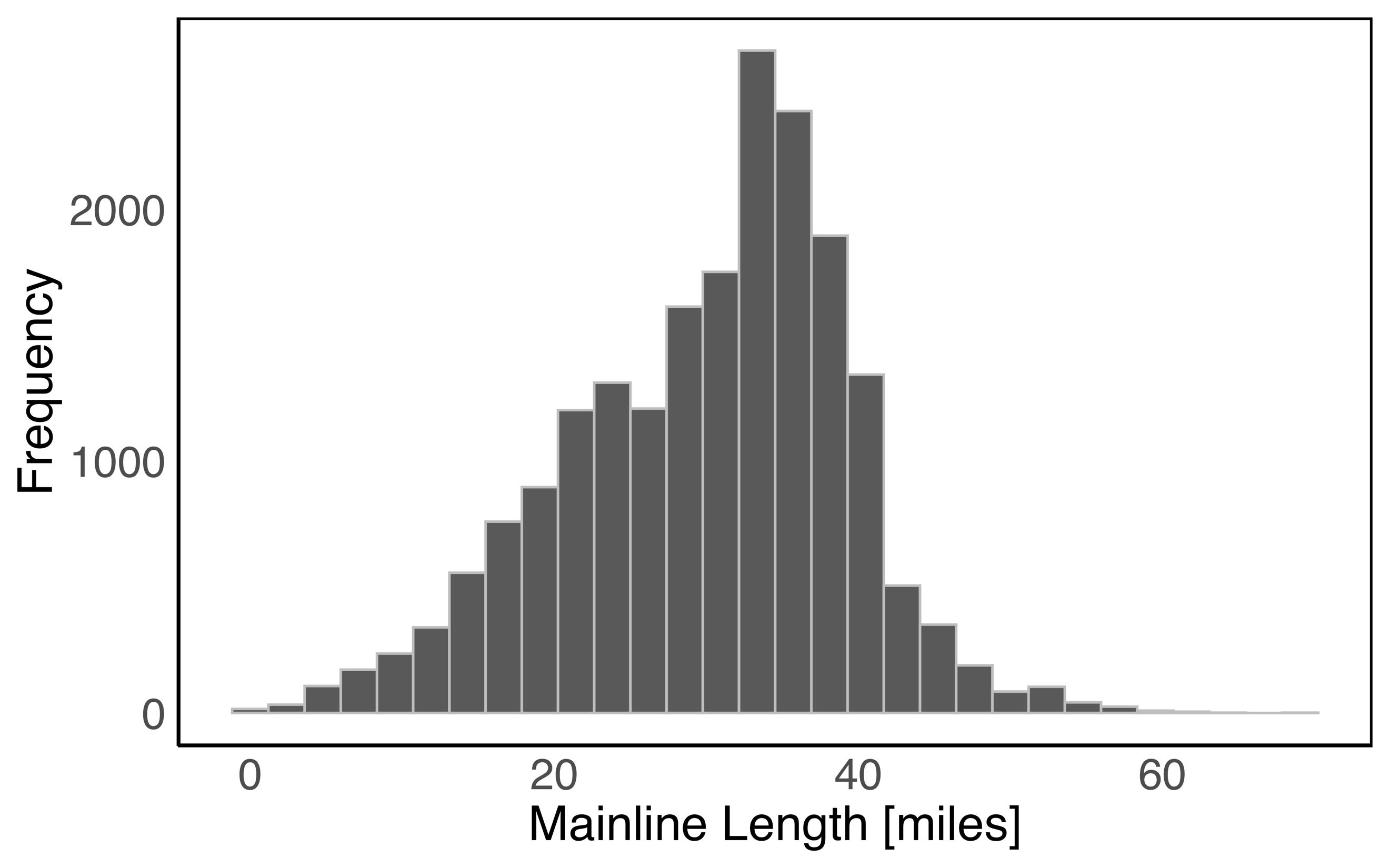


**Fig. S3**
